# Depressive symptoms and related factors in elderly diabetic patients

**DOI:** 10.1192/j.eurpsy.2021.635

**Published:** 2021-08-13

**Authors:** R. Gniwa Omezzine, W. Bouali, A. Belguith Sriha, L. Zarrouk

**Affiliations:** 1 Department Of Family Medicine. Tunisia., Monastir Faculty of Medicine, Mahdia, Tunisia; 2 Department Of Psychiatry, University Hospital Of Mahdia, Tunisia., Psychiatry, Mahdia, Tunisia; 3 Department Of Community Medicine, Monastir Faculty of Medicine, Mahdia, Tunisia

**Keywords:** diabetes, depression, elderly

## Abstract

**Introduction:**

Diabetes is a major public health problem in Tunisia. Its prevalence increases with age. In addition, depression, at the top of mental disorders list, mainly remain undiagnosed, in particular in the elderly and consequently untreated.

**Objectives:**

The aim of this study was to estimate depressive symptoms and related factors in elderly diabetic patients.

**Methods:**

This is a cross-sectional study, conducted among type 2 diabetic patients aged ≥ 60 years old, attending Mahdia’s primary health center, from January 2019 to March 2019. Depressive symptoms were assessed by using the Geriatric Depression Scale (GDS).

**Results:**

95 diabetic patients were recruited. The average age was 75 ± 7.4 years and the sex ratio was 0.9. In our sample, 68.4% of patients were categorized according to having depressive symptoms. The proportion of participants with mild and severe depression symptoms were 25.3% and 43.1%, respectively. Analytical results demonstrate many factors which were significantly associated with depressive symptoms: female gender, living alone, history of hypertension, presence of complication, and using insulin (p < 0.05).

**Conclusions:**

Our study shows that depressive symptoms are common in elderly subjects with diabetes, and there have been many significant risk factors associated with it. So there is need for physicians to detect, confirm, and treat depression in elderly diabetic patients.

